# A late diagnosis of hyperhomocysteinemia with probable evolution to verrucous elephantiasis nostra and leg ulcers^☆^^☆☆^

**DOI:** 10.1016/j.abd.2020.04.014

**Published:** 2021-01-31

**Authors:** Beatrice Martinez Zugaib Abdalla, Renan Tironi Giglio de Oliveira, Rafaela Issa Afonso, Paulo Ricardo Criado

**Affiliations:** a Faculdade de Medicina do ABC, Santo André, SP, Brazil; b Department of Dermatology, Faculdade de Medicina, Universidade de São Paulo, São Paulo, SP, Brazil; c Centro Universitário Saúde ABC, Santo André, SP, Brazil

*Dear editor,*

Homocysteine is an intermediate amino acid derived from the metabolism of methionine into cysteine, with a demonstrated association with oxidative stress and endothelial damage.^1^

Hyperhomocysteinemia can be caused by genetic disorders in metabolic pathways, nutritional deficiency, renal failure, hypothyroidism, diabetes, and smoking.^1^, ^2^, ^3^ It is an important risk factor for cardiovascular mortality in patients with a history of myocardial infarction, stroke, angina, diabetes, or hypertension.^2^

A black male patient, 60 years old, started follow-up with the dermatology service in 2011 due to symptoms of xerosis and lower limb edema in the last eight years. At the time, a biopsy was performed and revealed thickening of the epidermis, acanthosis, lengthening of the grooves, and deposits of dermal mucin. Colloidal iron staining was positive and Congo red staining was negative, the clinical and pathological diagnosis was pre-tibial myxedema.

The patient developed thrombophlebitis in the left lower limb, with no deep venous thrombosis. The authors decided to initiate antibiotic therapy for erysipela and, subsequently, prophylactic intramuscular benzathine benzylpenicillin was prescribed every 21 days. Topical care was performed with the use of dressings containning collagenase and chloramphenicol.

During evolution, periods of healing were interspersed with worsening. In 2018, when thrombophilia tests became available at the health institution, a panel for hypercoagulable states was performed: protein C and S dosages, antithrombin, total complement and C3, prothrombin gene, Leiden factor mutation, anticardiolipin and lupus antibodies, protein electrophoresis, and ANA, were all within the normal range. There were, serum homocysteine levels of 17.8 μmoL/L (normal range: 5 to 12 μmoL/L) and heterozygosity in segments C677 T and A1298C for the methylenetetrahydrofolate reductase (MTHFR) mutation was observed.

Another skin biopsy (Fig. 1) and Doppler ultrasonography of the left lower limb were performed. Histopathologically, there was fibroplasia, newly formed vessels associated with inflammatory lymphocytic infiltrate and a neutrophilic exudate in the dermis. Upon ultrasound examination, varicose veins, incontinence of the great saphenous, and popliteal veins associated with recanalized thrombophlebitis of the great saphenous vein were identified.

**Figure 1:**
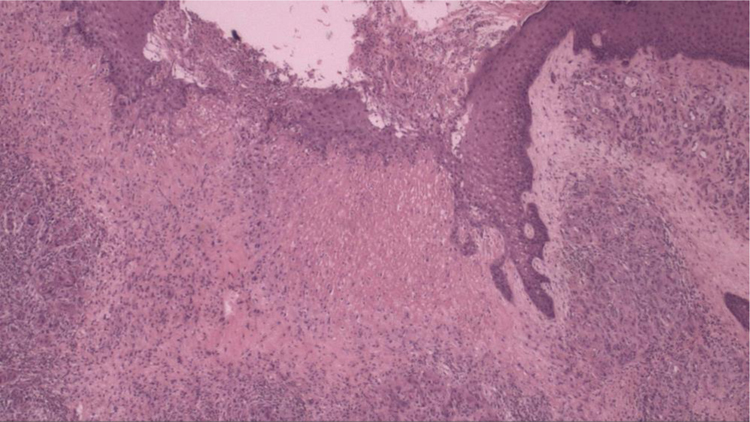
Histopathology demonstrating fibroplasia, newly formed vessels associated with an inflammatory infiltrate containing lymphocytes and neutrophils (Hematoxylin & eosin, ×40).

Treatment was initiated with the use of a vitamin B complex and oral folic acid, in addition to maintaining daily dressings.

After six months, serum homocysteine levels improved to 16.2 μmoL/L and the dermatological lesions healed (Figure 2, Figure 3).

**Figure 2:**
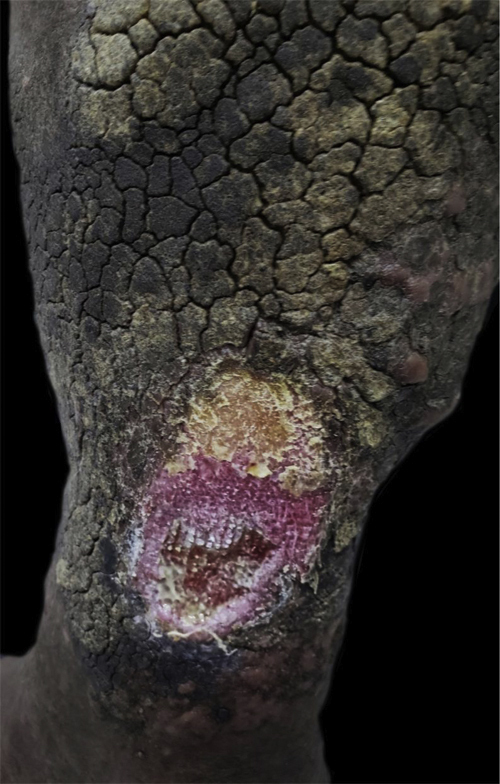
Left leg ulcer before treatment.

**Figure 3:**
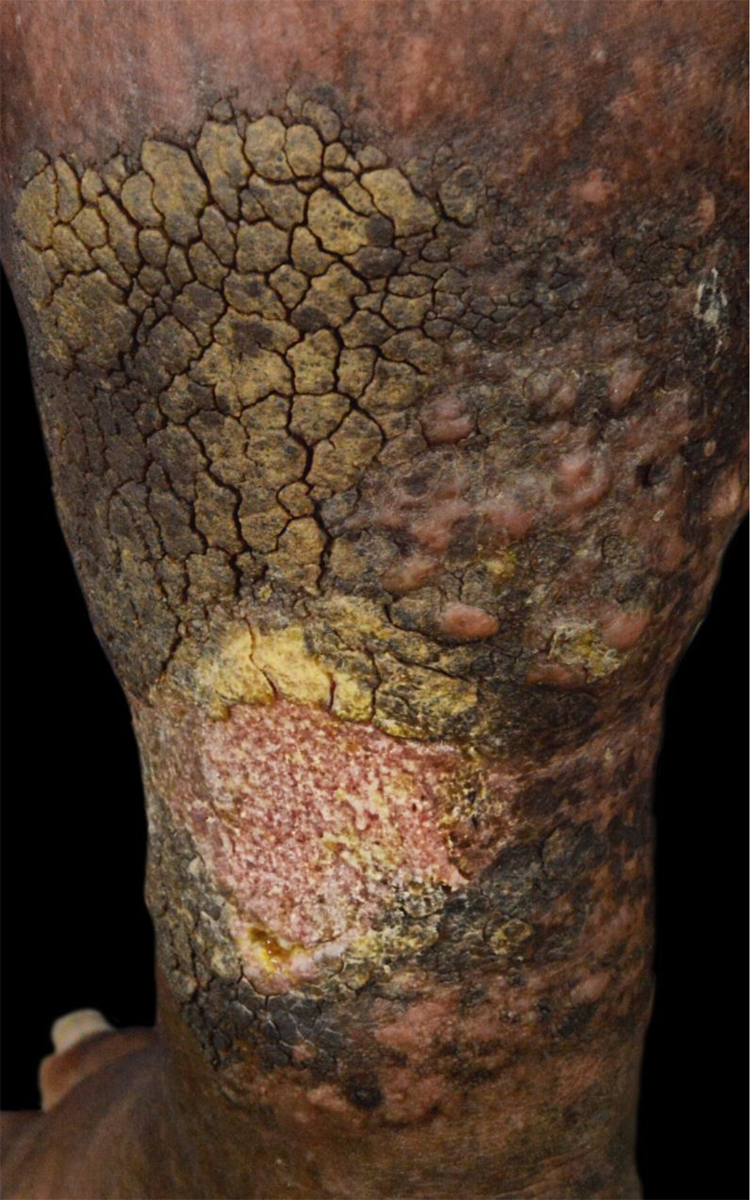
Complete healing of the left leg ulcer after two months of treatment.

In the subsequent one-year follow-up, the patient did not present new ulcers.

The vascular changes induced by hyperhomocysteinemia are multifactorial, including damage to the endothelium, increased lipid peroxidation, and platelet aggregation.^1^ The damage to the vessel is the result of an inflammatory process that causes the adhesion of neutrophils and T cells to endothelial cells, with subsequent release of cytokine IL-8 and monocytes-1 chemoattractant protein.^1^

The MTHFR enzyme catalyzes the methyltetrahydrofolate step necessary for the resynthesis of methionine from homocysteine.^3^ The cofactor vitamin B12 and folic acid participate in this metabolic pathway.^4^

The MTHFR gene has at least two functional polymorphisms, 677CT and 1298AC.^3^ The first allele is associated with reduced enzyme activity; concentrations in serum, plasma, and red blood cells; and increased plasma homocysteine concentration.^3^

Hyperhomocysteinemia is found in cases of dermatitis and ulceration due to stasis, which indicate that it may be associated with their pathogenesis.^1^, ^5^ Supplementation with vitamin B6, B12, and folic acid can decrease homocysteine levels, even in patients with normal serum vitamin concentrations.^1^, ^3^

This present case should alert physicians about hypercoagulable states in patients with leg ulcers under 50 years of age, as well as the need for a different approach in the evolution of lower extremity ulcers.

The authors advocate laboratory investigation of homocysteine in the differential diagnosis in cases of lower limb ulcers.^4^

The reduction of homocysteine using the replacement of certain selected vitamin supplements may be the future direction for preventing the development of the disease.^1^, ^3^

## Financial support

None declared.

## Authors’ contributions

Beatrice Abdalla: Approval of the final version of the manuscript; design and planning of the study; drafting and editing of the manuscript; collection, analysis, and interpretation of data; critical review of the literature.

Renan Tironi Giglio de Oliveira: Drafting and editing of the manuscript.

Rafaela Issa Afonso: Intellectual participation in propaedeutic and/or therapeutic conduct of studied cases.

Paulo Ricardo Criado: Approval of the final version of the manuscript; intellectual participation in propaedeutic and/or therapeutic conduct of studied cases; critical review of the literature; critical review of the manuscript.

## Conflicts of Interest

None declared.
